# Importance Assigned to Breastfeeding by Spanish Pregnant Women and Associated Factors: A Survey-Based Multivariate Linear Correlation Study

**DOI:** 10.3390/nu16132116

**Published:** 2024-07-02

**Authors:** Socorro Arnedillo-Sánchez, Jose Antonio Suffo-Abouza, Miguel Ángel Carmona-Rodríguez, Rubén Morilla-Romero-de-la-Osa, Inmaculada Arnedillo-Sánchez

**Affiliations:** 1 Department of Nursing, Faculty of Nursing, Physiotherapy and Podiatry, Universidad de Sevilla, 41009 Seville, Spain; marnedillo@us.es (S.A.-S.); jsuffo@us.es (J.A.S.-A.); migcarrod@alum.us.es (M.Á.C.-R.); 2 Midwifery Training Unit, Department of Materno-Fetal Medicine, Genetics and Reproduction, Hospital Universitario Virgen del Rocio, 41013 Seville, Spain; 3 Institute of Biomedicine of Seville, Hospital Universitario Virgen del Rocío/Consejo Superior de Investigaciones Científicas/Universidad de Sevilla, 41013 Seville, Spain; 4 Centro de Investigación Biomédica en Red de Epidemiología y Salud Pública (CIBERESP), 28029 Madrid, Spain; 5 School of Computer Science & Statistics, O’Reilly Institute, Trinity College Dublin, College Green 2, D02 PN40 Dublin, Ireland; macu.arnedillo@tcd.ie

**Keywords:** breast feeding, antenatal care, health education, maternal health, observational study

## Abstract

Breastfeeding education, across all disciplines, is often inconsistent and lacking in expertise and confidence. However, recommendations from health professionals, the sociocultural environment, and previous knowledge and experiences significantly influence women’s decision to breastfeed. This study aimed to identify factors that promote the assignment of greater importance to breastfeeding and associated practical benefits. This retrospective cross-sectional study included 276 participants who completed a self-administered questionnaire. Descriptive and bivariate analyses were performed, and multivariate linear models were applied to identify factors influencing the importance assigned to breastfeeding. Most participants were married or in a relationship, were native Spaniards, had secondary or higher education, and had an average age of 32.6 years. Seventy percent met the physical activity recommendations, and 91% felt comfortable with their body image during pregnancy. The importance assigned to breastfeeding was high across various aspects, except for postpartum weight loss and body image. Group prenatal care was only significantly associated with the importance assigned to the breastfeeding technique (how to breastfeed). The obesogenic environment and the importance assigned to nutritional aspects and physical activity also turned out to be predictors, although not for all models. In our region, the educational strategy of antenatal care groups could contain gaps regarding the mother’s health, which should be addressed in the future to improve results regarding the initiation and continuation of breastfeeding.

## 1. Introduction

Pregnancy is a key opportunity to reach women with essential services for their own health and that of their unborn child [1]. Antenatal care (ANC) is a careful, systematic assessment and follow-up of a pregnant women that includes education, counselling, screening, and treatment to assure the best possible health of the mother and her child [2]. Antenatal care (ANC) is crucial for protecting the health of mothers and children [3].

Education constitutes a vital aspect of prenatal care, especially for primigravid women. Pregnant women should be offered information based on the current available evidence, together with support to enable them to make informed decisions about their care [4]. ANC offers an essential setting for dialogue between pregnant women and healthcare providers (HCP) concerning health behaviors. HCP should offer information on postpartum and newborn care, breastfeeding practices, signs of alarm, and the appropriate course of action [5]. This education imparted during the antenatal period varies extensively in terms of delivery methods and content. It may enclose structured antenatal classes conducted in group or individual settings [6].

An essential part of ANC education is breastfeeding (BF) education. BF is widely acknowledged as the optimal nutritional source for infants [7]. It has been associated with enhanced child health, maternal health, and mother–infant bonding [8]. WHO and UNICEF recommend that BF be initiated within the first hour after birth and continued exclusively for the first six months of life and with safe and adequate complementary foods for up to 2 years or beyond [9]. The American College of Obstetricians and Gynecologists supports breastfeeding due to its benefits for the health of the mother and child, considering it a public health asset due to the health benefits it brings to the mother and child, with minimal contraindications to this practice [10].

Antenatal BF education is characterized by the provision of information during pregnancy. This may occur on an individual or group basis, including home visiting programs, peer education initiatives, and clinic appointments, and it may or may not involve partners. BF education typically entails a structured, defined, descriptive, and goal-oriented program designed for a specific purpose and audience [11].

It is important to acknowledge that HCP’s BF education, in all disciplines, is often inconsistent, lacking in expertise and confidence [12,13,14,15,16]. This can reduce the effectiveness of this type of educational intervention, and therefore, support tools are developed for providers who have shown statistically significant improvements in the intention and duration of breastfeeding [15]. Hospital counselling on breastfeeding showed a positive association with the initiation of this practice (aOR = 2.7, 95% CI = 1.60–3.96) that was significant across all races/ethnicities, which is a survey-based study conducted in Los Angeles [17].

However, the recommendations of HCP influence pregnant women’s decision to breastfeed [18]. Scientific evidence has been reported of the effectiveness of counselling in the initiation of breastfeeding depending on the type of provider. Thus, advice from midwives and obstetricians is more effective than that from other specialists. Additionally, women cared for by midwives were 68% less likely to never breastfeed than those cared for by an obstetrician [19].

BF intention, attitude, and knowledge have been shown to be strong predictors of initiation [20,21,22]. In Saudi Arabia, sociodemographic factors have been identified as predictors; housewives were four times more likely to have a high EBF intention compared to working mothers [aOR = 4.216 (1.906–9.326), *p* < 0.001]. Similar results for housewives were reported in a longitudinal study carried out with women form Lebanon and Qatar [21]. Having adequate knowledge and a positive attitude towards breastfeeding were also positive predictors ([aOR = 1.193 (1.183–1.421), *p* = 0.009] and [aOR = 1.282 (1.205–1.365), *p* = 0.000], respectively) [20]. Women who were encouraged to breastfeed have more chances of BF compared to those who did not receive this support [11]. As per the conclusions drawn from a Cochrane review, provider encouragement led to an increase in exclusive BF at 3 months and a prolonged BF duration up to 4 months [23].

Despite the many advantages and extensive promotion of BF, the trend towards BF in many countries has been slowly increasing. Globally, the prevalence of exclusive BF during the initial six months of life has risen by 10% over the last decade, reaching 48% in 2023 and nearing the World Health Assembly’s objective of 50% by 2025 [24].

It has been shown that analyzing the importance that women give to breastfeeding based on their knowledge and previous experiences, both for its initiation and continuation, should be an object of study at the regional level due to possible sociocultural influences and those specific to the healthcare systems. However, these aspects remain unclear in our region.

The aim of this study was to determine how the sociodemographic, dietary, and physical activity profiles of pregnant women, in our demographic context, may influence the importance they assign to different aspects of BF. Additionally, after the participation in group ANC, the efficacy of the disclosed messages regarding BF was evaluated by the significance women assigned to them. These included postpartum weight management, the prevention of childhood obesity, the infant’s overall health, as well as the factors influencing their prioritization. Through this analysis, our aim was to enhance the understanding of the influence of BFE in group ANC on pregnant women’s importance assigned to BF, with the potential to detect strengths and gaps in the content of BF education in our context.

## 2. Materials and Methods

### 2.1. Research Design and Participants

This was an observational, retrospective, cross-sectional study. The study was conducted according to the latest Consolidated Standards of Reporting Observational Studies (STROBE) 2008 guidelines for reporting observational studies [25].

The study was conducted in a Spanish third-level hospital that cares for 5196 births annually [26]. Woman of all ages and socioeconomic background attend the hospital, which offers high complex services as well as basic obstetric care. Women receive ANC through the Public Health System, which encompasses both primary care (midwife and General Practitioner) and obstetric assistance. BF education is integrated into ANC, where the intention towards BF is documented in health records from the first individual visit. BF education primarily occurs within group ANC in the third trimester. This session covers aspects including the anatomy, the benefits of BF, BF techniques, BF positions, breast milk expression, myths surrounding BF, contraception, as well as the disadvantages of formula feeding [27]. Despite recommendations by health authorities, midwives can arrange their BF education according to their own preferences. The hospital is in the third phase of the Mother Baby Friendly Hospital accreditation. At admission for delivery, all women are asked about plans for infant feeding. Skin-to-skin contact and BF is initiated immediately after vaginal delivery. When the clinical condition of the mother is stable after a caesarean section, BF is initiated within 120–180 min of delivery. In addition, all women who give birth in the hospital are given practical BF training by midwives, nurses, and pediatricians, BF is observed, and support and BF counselling services are provided.

A consecutive sampling procedure was employed to select participants. To determine the minimum sample size required for this study, Gpower software v3.1 (t-ref version) was used. The following parameters were selected: family test (F-Test), statistical test: Linear multiple regression: fixed model, R^2^ deviation from zero. The type of analysis was as follows: the required sample size-given alpha (0.05), power (0.95), and effect size (0.1) were computed. No similar studies were found to determine a theoretical effect size, so a minimum effect size was employed to maximize the sample size. The minimum number of participants to include in the study was 204.

The study´s inclusion criteria were adult (≥18 years old) singleton pregnant women at ≥37 gestational weeks who accepted and signed the informed consent form. The exclusion criteria were pregnant women with mental instability and/or communication difficulties due to language barriers or who were not willing to participate.

### 2.2. Data Collection Methods and Survey Period

Women were recruited during a prenatal visit to the hospital. The study’s objectives and aims were explained by a midwife researcher. A self-administered questionnaire was designed following a literature review. It consisted of three main sections: the socio-demographic and obstetric profile, lifestyle habits, and the importance assigned to different aspects of BF.

Sociodemographic and obstetric data included age, place of birth, education, civil status, obesogenic environment, parity, attendance to group ANC, and smoking.

Lifestyle habits included eating habits during pregnancy, physical activity, and adherence to a Mediterranean diet (MD). Appropriate Physical activity was considered following WHO recommendations (at least 150 min of moderate-intensity aerobic physical activity throughout the week) [28]. Adherence to MD was accessed through the MEDAS scale [29]. It consists of 14 items, where participants report their habitual frequency or amount consumed of 12 main components of MD, along with two food habits related to MD. Each of the items scored either 1 or 0 based on participants’ adherence. The resulting MD score derived from MEDAS ranges from 0 to 14 [30].

The participants were asked to indicate the importance they assigned to different aspects of BF through a Likert scale, with five-point response options ranging from Not at all important (1) to Very important (5). These aspects included the BF technique, BF and postpartum weight loss, BF and baby’s health, and BF and childhood obesity.

The survey was pilot-tested for clarity by 50 respondents. The data collection took place between March and December 2019. Pregnant women completed the questionnaire during a prenatal visit, and anthropometric data were retrieved from the Pregnant Women´s Health document.

### 2.3. Data Analysis

Statistical analyses were performed using SPSS software (version 23; IBM Corporation, Armonk, NY, USA). Descriptive analyses were performed with the use of frequencies and measures of central tendency and dispersion to characterize the study sample and the results of the questionnaire. To identify factors associated with the importance assigned to BF, bivariate analyses were performed by chi-square analysis and Fisher’s exact test for qualitative variables, where appropriate, and parametric or non-parametric tests of mean comparison according to normality and homoscedasticity criteria [31]. To evaluate the potential confounder effects of the variables that reached statistical significance, multivariate linear models were performed to estimate what factors influence the importance women assigned to different aspects of BF. We followed a rule of thumb commonly used in statistics and regression modeling that suggests that, at a minimum, you should have 10 events per variable to adequately estimate the model parameters. In the context of multivariate linear regression, this would translate into having at least 10 subjects for each covariate in your model. This allowed, according to our sample size, to include 26 covariates in initial models, and the Backward Elimination technique was applied (the covariates are iteratively eliminated one by one, starting with the one with the highest *p* value, that is, the least significant statistically). In both bivariate and multivariate analyses, statistical significance was set at 5%.

### 2.4. Ethical Aspects

Ethics committee approval was obtained from the Ethics Committee (Protocol C.P. MSA-FP-2019-01-C.I.). Written and verbal informed consent was obtained from the participants. The participants signed an informed consent form declaring their voluntary participation once they understood the objectives and dynamics of the study. They were aware of the possibility of abandoning the study at any time and that the processing of their information would be anonymous and under the precepts of current legislation on the processing of personal data.

## 3. Results

Two hundred and seventy-six (276) participants responded to the questionnaire; however, not all women answered all questions. Table 1 and Table 2 show missing data for each item. The profile of women was mainly native Spaniards who were married or in a relationship, with secondary or high education and an average age of 32.6 (SD 5.8), ranging between 18 and 47 years old. Regarding physical activity, 70% of the sample met WHO physical activity recommendations for pregnant women. Only 9% felt uncomfortable with their body image during pregnancy. Table 1 shows data for sociodemographic obstetric characteristics and physical activity (Table 1).

Almost half of the women had a healthy pregestational BMI, most did not have a gestational weight gain (GWG) target, and GWG was evenly distributed among its three categories, inadequate, healthy, and excessive. Over half of the sample changed their eating habits, maintaining the same appetite and food amount. Half presented craving and 60% presented nauseas. Only 18% of the sample had a low adherence to MD (Table 2).

Table 3 shows the importance assigned to different aspects through a Likert scale, with higher punctuations for more importance assigned. The average punctuation for all variables was over four out of five points, except the importance assigned to BF for postpartum weight loss and body image before and during pregnancy (near four points). More than half of the participants had a good adherence to MD, with a median score 9.3 over 14 points.

Four multivariate linear regression models were evaluated, in which those variables related to BF and different aspects such as the BF technique and importance as a protective factor against childhood/maternal obesity and as a guarantor of the baby’s (general) health were included as dependent variables (Table 4).

The first aimed at seeing what factors influenced the importance assigned by mothers to the BF technique. It showed that the presence of obese subjects in the family environment, the importance assigned to nutrition during BF, BF as a guarantor of the baby´s health, as well as attendance to group ANC were favorable factors, although they had little impact. However, the model overall obtained a coefficient of determination of 0.62, resulting in a significant model.

For the model that evaluated the importance assigned to BF as a tool for losing weight in the postpartum, obesity in the family environment, concern about BI before pregnancy, and participants that assigned a high importance to BF as a tool for preventing childhood obesity were observed as factors with a direct relationship. However, a statistically significant association was observed, indicating an inverse relationship with non-smoking behaviors (neither prior to nor during pregnancy).

The third model investigated the importance assigned to BF as a protective factor against childhood obesity. In it, the importance assigned to physical activity during pregnancy, the BF technique, and BF as a tool for losing weight in the postpartum period appeared as promoting factors.

Finally, the last model assessed the importance assigned to BF as a means of maintaining the baby’s health. A significant direct relationship was observed with the importance assigned to the BF technique and to BF as a tool for preventing childhood obesity. However, it was inversely related to the presence of an obesogenic environment. Negative coefficients also resulted for considering physical activity during pregnancy as negative for the baby’s health, one’s health, or neither, using the health of both (mother and baby) together as a reference category.

## 4. Discussion

The result of this study suggests the effectiveness of information anchoring regarding the BF technique obtained during group ANC, identifying this moment as a key time to include additional information regarding BF that could mediate in women´s decisions regarding BF, initiation, and maintenance rates. It highlights some factors that promote the importance women assign to BF in relation to benefits for both mothers and babies. Additionally, it is a descriptive study on sociodemographic, dietary, and physical activity during pregnancy in women from our environment. These factors were explored as possible promoters of the importance assigned to the discussed issues, with no significant results.

BFE places much emphasis on the BF technique, which is crucial for BF initiation and maintenance [32]. The findings of this study suggest that certain aspects concerning BF, like its influence on maternal health, are being inadequately addressed, potentially hindering pregnant women from recognizing their significance. This is evident in the models obtained, as attendance to group ANC only emerged as a promoting factor for the importance assigned to the BF technique. Although women attribute medium to high importance values (above three) to the rest of the issues (Table 2), attendance to group ANC did not appear as a promoting factor in the respective models. Considering the positive effect that group ANC had on the BF technique message, it would be clinically interesting to include and promote other aspects. Given the importance that postpartum weight retention has on the obesity epidemic, [33,34], as well as the negative consequences of childhood obesity [35], these aspects should be emphasized.

Although our findings indicate pregnant women are concerned about BF techniques, a qualitative study among primary care Spanish midwives revealed that many women attending group ANC tend to prioritize discussions on delivery and associated pain, often relegating BF to a secondary concern [36]. This aligns with a recent study in first-time mothers in England, who indicated that obtaining trustworthy information through direct interactions with experts was essential for meeting their needs and boosting their confidence. However, when questioned about the type of information that would serve this purpose, their attention was only directed toward childbirth [37]. Paz-Pascual noted that Spanish women often expressed a preference for exclusive BF to be addressed preferably in postnatal classes, alongside topics like newborn care and motherhood, which we believe would be too late. They also manifested the need for greater accompaniment in the puerperium and less pressure concerning BF [38]. In this line, women identified themselves and society (HCP, media, and partners) [39] as the main sources of pressure to BF. This pressure was negatively associated with the BF experience (r = −0.34, *p* < 0.01) and self-efficacy (r = −0.39, *p* < 0.01) [40], as well as with mental health symptoms [39]. This leads us to consider that although BF support is essential in the puerperium, it could be interesting to seek a more suitable time during pregnancy to address BF. We suggest that during the second trimester, women are less focused on childbirth and could therefore be more receptive to BFE. Supporting this proposition, some authors have described that maternal concerns and pregnancy-related anxiety are more intense in early and late pregnancy [41,42]. We also believe that it is necessary to implement educational strategies that, while promoting BF as the optimal way to feed babies, relieves women from the pressure they feel.

The BF rates among participants in group ANC, when compared to individual care, suggest that group ANC shows significantly higher rates of BF initiation and exclusive BF at hospital discharge [43,44]. Gray (2024) reported that it increases BF at 6 months (odds ratio = 2.66; *p* = 0.045) and leads to higher BF intention, competence, and autonomous motivation initiation [45]. The Andalusia Birth and Parenting Preparation Guide [27] recommends that BF be addressed during one group ANC visit, along with individual care. However, we believe that a single visit may not sufficiently raise mothers’ awareness about the importance of BF beyond the baby’s health. Effective BFE requires more attention to significantly impact mothers’ awareness of its importance. Nasim reported a dose-response effect; more ANC visits had higher rates of BF initiation and durations [46]. Stakeholders should take note of these findings and ensure that ANC includes the appropriate number of HCP and visits. It is worth noting that Spain has one of the lowest rates of midwives in the European Union, with 17–21 practising midwifes per 100,000 inhabitants [47] and a currently understaffed workforce. Midwife care has been associated with better health results for mothers and children, including higher BF rates [48,49,50]. Likewise, insufficient support from the health system reduces the probability of BF [13].

In our study, women assigned a lot of importance to BF in relation to the baby´s health and BF technique, while the importance regarding the mother´s health and its influence on puerperal weight loss had the least importance. This highlights that pregnant women seem more concerned about their babies than themselves. We suggest, in line with other authors, that ANC should embrace new perspectives that approach holistic maternal health issues, not solely focusing on the child´s care [51]. A reorientation towards a health promotion that is mother-centred is crucial to meet women´s needs and enhance health results [52]. Greater emphasis could be placed on women’s decision to breastfeed and its short- and long-term benefits for both the mother and newborn.

Despite the fact that, as our results confirm, in line with other research [53], most women felt comfortable or reported to look beautiful during pregnancy, body dissatisfaction during the postpartum is common [54]. Expectations of the ideal Western beauty standard, mediated by social norms, pressure mothers to quickly return to pre-pregnancy weight soon after birth [55]. This is a goal that is often not achieved. Dissatisfaction with the maternal body is linked to several negative outcomes, including postpartum depression [56,57] and shorter BF durations [58]. In our model, women who assign more points to the importance of BF as a tool for recovering pregestational weight also do so to the importance of BF for the prevention of childhood obesity. Promoting these positive aspects of the BF experience in ANC could serve as a catalyst for encouraging the early initiation and maintenance rates of BF and its associated benefits. For example, we strongly believe that BFE should highlight the importance of BF as a mediator of maternal health, including negative outcomes related to body dissatisfaction related to postpartum weight retention.

Our results show that adherence to MD in our sample was high, with better results than those reported for Italy for pregnant women [59]. No association between adherence to MD and importance assigned by pregnant women to different health-related aspects was found. However, the MD pattern is associated with benefits during pregnancy and the prevention of certain diseases, including cardiovascular diseases [60]. Its promotion among pregnant women could serve as a beneficial public health strategy for preventing overweight and nutrient deficiencies [61]. Research also suggests that women who choose to BF tend to follow healthier diets, have better dietary knowledge, and promote an overall healthier diet in their children [62]. Breastfeeding for 6 months or longer is associated with a higher adherence to the MD during the preschool years [63]. Women who adhered to the MD and breastfed not only achieved physical benefits such as a lower proportion of corporal fatty tissue [64] but also achieved emotional benefits such as a lower risk of depressive episodes [65]. Due to the benefits that MD has on mothers and newborns in the long and short term, its promotion should be a fundamental aspect in ANC. While mastering BF technique is crucial for new mothers to initiate and sustain lactation, BFE should also emphasize maternal health benefits, incorporate body image concerns, and promote MD [66].

We suggest that our results are interesting for HCP and midwives in our context, highlighting the need to shift BFE towards a more women-centered approach, employing pedagogic models grounded in principles of adult learning. Such education should furnish women with realistic, detailed, and positively affirming information.

This study has some limitations. First, the design, an observational study, while serving as a useful means to establish prevalence rates and identify risk factors, may not offer the strongest scientific evidence. However, it has been demonstrated to address knowledge gaps and provide information needed to improve decision-making. Second, the use of non-randomized sampling could introduce bias. Nonetheless, we believe that employing multivariate analysis helped mitigate this issue. Although researchers were aware of the recommendations for group ANC and BFE by health authorities, they did not have information on the specific content and duration of BFE.

## 5. Conclusions

The variability explained by our models ranges between, approximately, 46% and 66%, which represents successful results. Through the analysis of the importance Spanish pregnant women assigned to different aspects of BF and care during pregnancy, we suggest that group ANC is effective in anchoring information regarding the BF technique. However, attendance at group ANC did not appear as a predictor in other importance assignment models, which could imply that the education received contains gaps that must be addressed. In this sense, BF education often overlooks maternal health benefits. The obesogenic environment appeared as a positive predictor in the importance assigned to how to BF and breastfeeding as a tool for losing post-partum weight. However, it was a negative predictor for the importance assigned to BF in maintaining the baby’s health.

Although the importance assigned to nutrition was a predictor of how to breastfeed, pregestational BMI, adherence to a Mediterranean diet, and sociodemographic features did not appear as predictors in any model.

Since, in our region, breastfeeding education has long emphasized the breastfeeding technique, as has been positively reflected in the results, antenatal BF education should be complemented with other important aspects related to breastfeeding so that women benefit from this information—for example, regarding maternal health.

## Figures and Tables

**Table 1:** Sociodemographic and obstetric characteristics and physical activity.

Variables	Total Women		% (IC 95)
	*N*	*n*	
Nationality	263		
Spanish		247	93.9 (90.4–96.2)
Eastern Europe		5	1.9 (0.8–4.4)
Hispa-American		10	3.8 (2.1–6.7)
Others		1	0.4 (0.1–2.1)
Educational level	275		
Higher Education		109	39.6 (34.0–45.5)
Primary School		42	15.3 (11.5–20.0)
Secondary School		116	42.2 (36.5–48.1)
None		8	2.9 (1.5–5.6)
Civil Status	274		
Marriage/couple		261	95.2 (92.1–97.2)
Single		5	1.8 (0.8–4.29)
Others		8	3 (1.5–5.7)
Nullipara	276	174	63 (57.2–68.5)
Smokes in pregnancy	275	31	11.3 (8.1–15.6)
Obesogenic Environ.	274	40	14.6 (11.0–19.3)
Group Antenatal Care	273	193	70.7 (65.0–75.8)
Pg Physical Activity	272	160	59 (53.0–65.0)
Info Physical Activity	271	213	78.6 (73.3–83.1)
Believes PA negative	269	28	10.4 (7.3–14.6)
Changes in PA	255	149	58.4 (52.3–64.3)
Meets WHO PA recom.	215	151	70.2 (63.8–76.0)
APP PA	243	44	18.1 (13.8–23.4)
Pregnancy body Image	275		
Pretty		133	48.4 (42.5–54.3)
Neither pretty nor ugly		115	41.8 (36.1–47.7)
Uncomfortable		27	9.8 (6.8–13.9)

Note: Environ, environment; Pg, pre-gestational; PA, Physical Activity; WHO, World Health Organization; recom, recommendation; APP, application.

**Table 2:** Anthropometrics and eating habits.

Variables	Total Women		% (IC 95)
	*N*	*n*	
pgBMI	275		
Underweight		7	2.6 (1.2–5.2)
Normal		127	46.2 (40.4–52.1)
Overweight		84	30.5 (25.4–36.2)
Obesity		57	20.7 (16.4–26.0)
Gestational Weight Gain	275		
Inadequate		87	31.6 (26.4–37.4)
Healthy		91	33.1 (27.8–38.9)
Excessive		97	35.3 (29.9–41.1)
GWG Target	267		
Increase		11	4.2 (2.4–7.4)
Maintain		63	24 (19.4–29.7)
Lose		2	0.8 (0.2–2.8)
None		185	71 (65.1–76.1)
Eating Habits Change	264	142	53.8
Eat for two	267		
No		253	94.8 (91.4–96.9)
Appetite	139		
Increased		17	12.2 (0.8–1.9)
Same		73	52.5 (4.4–6.1)
Decreased		49	35.3 (2.8–4.3)
Change in Quantity	273		
More		85	31.1 (2.6–3.7)
Same		139	51.0 (4.5–5.8)
Less		49	17.9 (1.4–2.3)
Snacking	270	162	60 (54.1–65.7)
Stress/anxiety	267		
Eat more		119	44.6 (3.9–5.1)
Eat the same		97	36.3 (3.1–4.2)
Eat less		51	19.1 (1.5–2.4)
Craving	270	132	49 (43.0–55.0)
Nausea	270	169	62.6 (56.5–68.2)
Adherence to MD	216	155	71.8 (65.4–77.3)

Note: pgBMI, Pre-gestational Body Mass; GWG, Gestational Weight Gain; MD, Mediterranean Diet.

**Table 3:** Importance assigned to aspects of Breastfeeding and Descriptive Quantitative Variables.

Variable	Media	SD	Minimum	25%	50%	75%	Maximum	*n*	NA
ADH_MD.9	9.3	2.1	1.0	8.0	10.0	11.0	14.0	216	60
Age	32.6	5.8	16.0	29.0	33.0	37.0	47.0	276	0
Image Before	3.7	1.0	1.0	3.0	4.0	4.0	5.0	272	4
Image Pregnancy	3.5	1.1	1.0	3.0	4.0	4.0	5.0	271	5
pgBMI	26.1	5.7	17.3	22.1	25.0	28.3	46.0	275	1
Importance PA	4.1	1.1	1.0	3.0	4.0	5.0	5.0	250	26
Importance Nut	4.3	1.1	1.0	4.0	5.0	5.0	5.0	253	23
Importance BF How	4.6	0.8	0.0	5.0	5.0	5.0	5.0	273	3
Importance BF PCO	4.1	1.2	2.0	3.0	5.0	5.0	5.0	246	30
Importance BF PWL	3.7	1.3	2.0	3.0	4.0	5.0	5.0	246	30
Importance BF BH	4.7	0.8	0.0	5.0	5.0	5.0	5.0	273	3

Note: SD, standard deviation; NA, not available; ADH_MD, Adherence to Mediterranean Diet; BMI, Body Mass Index; PA, Physical Activity; Nut, Nutrition; BF, Breast Feeding; PCO, Prevent Child Obesity; PWL, Postpartum Weight Loss; BH, Baby´s Health.

**Table 4:** Lineal models.

Model 1: Dependent Variable: IA to How to BF			
Independent Variables	Estimate	*p*-Value	Models’ Parameters
Intercept (beta 0)	0.40	0.067	Adjusted R^2^: 0.62 F-statistic: 98.03 on 4 and 239 DF, *p*-value: <0.001
Obesogenic Environment	0.24	0.006	
Importance Nut	0.14	<0.001	
Importance BF BH	0.74	<0.001	
Group Antenatal Care	0.20	0.003	
**Model 2: Dependent Variable: IA to BF as a Tool for Losing Post-Partum Weight**			
**Independent Variables**	**Estimate**	***p*** **-Value**	**Models’ Parameters**
Intercept (beta 0)	0.73	0.024	Adjusted R^2^: 0.48 F-statistic: 46.02 on 4 and 227 DF, *p*-value: <0.001
No pregnancy smoking	−0.31	0.021	
Obesogenic Environment	0.33	0.057	
Image Pregnancy	0.14	0.036	
Importance BF PCO	0.65	<0.001	
**Model 3: Dependent Variable: IA to BF as a Preventive Factor of Childhood Obesity**			
**Independent Variables**	**Estimate**	***p*** **-Value**	**Models’ Parameters**
Intercept (beta 0)	−0.06	0.87	Adjusted R^2^: 0.47 F-statistic: 65.8 on 3 and 219 DF, *p*-value: <0.001
Importance PA	0.19	0.004	
Importance BF How	0.31	<0.001	
IA to BF as a means of losing pp-weight	0.52	<0.001	
**Model 4: Dependent Variable: IA to BF to Maintain Baby’s Health**			
**Independent Variables**	**Estimate**	***p*** **-Value**	**Models’ Parameters**
Intercept (beta 0)	1.14	<0.001	Adjusted R^2^: 0.62 F-statistic: 64.02 on 6 and 227 DF, *p*-value: <0.001
Obesogenic environment	−0.20	0.022	
Importance BF How	0.76	<0.001	
Importance BF PCO	0.05	0.065	
Your health—baby’s [baby´s]	−0.39	0.042	
Your health—baby’s [main]	−0.78	0.029	
Your health—baby’s [none]	−0.18	0.051	

Note: IA, Importance assigned; BF, breast feeding; pp, post-partum.

## Data Availability

The original contributions presented in the study are included in the article, further inquiries can be directed to the corresponding author.
